# β-Elemene Attenuates Renal Fibrosis in the Unilateral Ureteral Obstruction Model by Inhibition of STAT3 and Smad3 Signaling via Suppressing MyD88 Expression

**DOI:** 10.3390/ijms23105553

**Published:** 2022-05-16

**Authors:** Wenjuan Sun, Dong Hyun Kim, Chang Hyun Byon, Hoon In Choi, Jung Sun Park, Eun Hui Bae, Seong Kwon Ma, Soo Wan Kim

**Affiliations:** Department of Internal Medicine, Chonnam National University Medical School, 42 Jebongro, Gwangju 61469, Korea; kathy66kao@163.com (W.S.); dhkim450@gmail.com (D.H.K.); changhyunbyon@gmail.com (C.H.B.); hoonin_c@hanmail.net (H.I.C.); gene-pjs@hanmail.net (J.S.P.); baedak76@gmail.com (E.H.B.); drmsk@hanmail.net (S.K.M.)

**Keywords:** renal fibrosis, β-elemene, STAT3, Smad3, Myd88

## Abstract

Renal fibrosis is a chronic pathological process that seriously endangers human health. However, the current therapeutic options for this disease are extremely limited. Previous studies have shown that signaling factors such as JAK2/STAT3, Smad3, and Myd88 play a regulatory role in renal fibrosis, and β-elemene is a plant-derived sesquiterpenoid organic compound that has been shown to have anti-inflammatory, anti-cancer, and immunomodulatory effects. In the present study, the anti-fibrotic effect of β-elemene was demonstrated by in vivo and in vitro experiments. It was shown that β-elemene inhibited the synthesis of extracellular matrix-related proteins in unilateral ureteral obstruction mice, and TGF-β stimulated rat interstitial fibroblast cells, including α-smooth muscle actin, vimentin, and connective tissue growth factor, etc. Further experiments showed that β-elemene reduced the expression levels of the above-mentioned fibrosis-related proteins by blocking the phosphorylation of JAK2/STAT3, Smad3, and the expression or up-regulation of MyD88. Notably, knockdown of MyD88 attenuated the phosphorylation levels of STAT3 and Smad3 in TGF-β stimulated NRK49F cell, which may be a novel molecular mechanism by which β-elemene affects renal interstitial fibrosis. In conclusion, this study elucidated the anti-interstitial fibrosis effect of β-elemene, which provides a new direction for future research and development of drugs related to chronic kidney disease.

## 1. Introduction

Chronic kidney disease (CKD) is one of the fastest-growing causes of death worldwide, with progressive loss of kidney function leading to end-stage renal disease (ESRD) [1]. Fibrosis is considered the ultimate common pathway leading to ESRD [2]. The pathogenesis of renal fibrosis is characterized by glomerulosclerosis, fibrosis of the tubular interstitium and blood vessels, and scar formation. It is worth mentioning that renal fibrosis is associated with extracellular matrix (ECM) deposition, decreased ECM degradation, and ECM protein cross-linking, leading to changes in the mechanical properties of the tissue and its response to noxious stimuli and progressive progression to ESRD [3,4]. It is well known that myofibroblasts and activated fibroblasts proliferate and produce large amounts of ECM that accumulate in the renal tubular interstitium; together with tubular atrophy, this accumulation leads to interstitial fibrosis [5]. A large body of evidence suggests that the expression of α-smooth muscle actin (α-SMA) in myofibroblasts and a variety of other fibrosis-associated cells is an important link in the process of ECM aggregation [6]. Vimentin and connective tissue growth factor (CTGF) are involved in cell proliferation, migration, and differentiation, and can directly contribute to the fibrosis process, both of which are expressed at low levels in normal renal tissues [7,8]. Meanwhile, the adhesive glycoprotein, fibronectin, is involved in the formation of ECM since the early stages of fibrosis, and its accumulation is an important event in the process of renal fibrosis [9].

Unilateral ureteral obstruction (UUO) is the classic model for studying interstitial fibrosis in the kidney. This model is based on the ligation of one ureter, which leads to urinary tract obstruction and triggers a series of rapid events [10]. Specifically, renal blood flow and glomerular filtration rate are significantly reduced within the first 24 h [11]. Continued ischemia and hypoxia lead to further hydronephrosis, interstitial inflammatory cell infiltration, and tubular cell apoptosis and necrosis, ultimately leading to progressive renal fibrosis [12,13].

The Smad family signaling cascade plays an important role in fibrosis. Smad3 is the most characterized member of this family, playing a key role in activating the myofibroblast phenotype, stimulating ECM synthesis, integrin expression, and secreting proteases and antiproteases [14]. Dysregulation of the TGF-β/Smad pathway may be a major mechanism of tissue fibrosis, and it is commonly believed that Smad3 directly promotes TGF-β mediated tissue fibrosis [15]. Specifically, upon activation by TGF-β, phosphorylated Smad3 is translocated to the nucleus, where it regulates the transcription of fibrosis target genes such as collagen I, α-SMA, and CTGF [16,17,18]. Notably, Smad3 signaling is active in diseased kidneys not only because of TGF-β upregulation but also because of dysregulation of other Smad-related repressors or regulators [19].

STAT3 is a polymorphic transcription factor involved in a range of physiological and pathophysiological processes, including inflammation, cell growth, proliferation, and differentiation [20,21,22,23]. STAT3 has been shown to induce the expression of multiple fibrotic genes and can transduce the pro-fibrotic effects of TGF-β from the cell surface to the nucleus. First, TGF-β promotes the accumulation of p-JAK2, which further phosphorylates STAT3. The phosphorylated STAT3 forms a dimer, which is then transferred to the nucleus to regulate the expression of fibrotic or pro-inflammatory genes [24,25,26]. Notably, several studies have shown that increased STAT3 activation is involved in UUO-induced renal fibrosis in mesenchymal fibroblasts [27,28].

Despite the known existence of multiple cellular and molecular mechanisms associated with renal fibrosis, there is still a lack of methods and drugs to inhibit or retard renal fibrosis. The potential of natural plant extracts as drug candidates have been widely recognized [29]. β-elemene, an extract of the rhizome of Curcuma longa, has been shown in several studies to have broad antitumor activity. It could inhibit tumor growth and proliferation, induce apoptosis, and modulate immunity [30,31,32]. β-elemene also exerts anti-inflammatory effects by inhibiting CCl_4_-induced serum TNF-α and endotoxin in rats, and experimentally demonstrated that β-elemene attenuates liver fibrosis [33]. However, the role and mechanism of β-elemene as reported in renal fibrosis remains unclear. In this study, we evaluated the anti-fibrotic activity of β-elemene in TGF-β treated rat interstitial fibroblast (NRK49F) cell and an established unilateral ureteral obstruction (UUO) mouse model. This adds new evidence for the therapeutic potential of β-elemene for future clinical applications.

## 2. Results

### 2.1. β-Elemene Ameliorated Histopathological Alterations in UUO Mice

**Figure 1:**
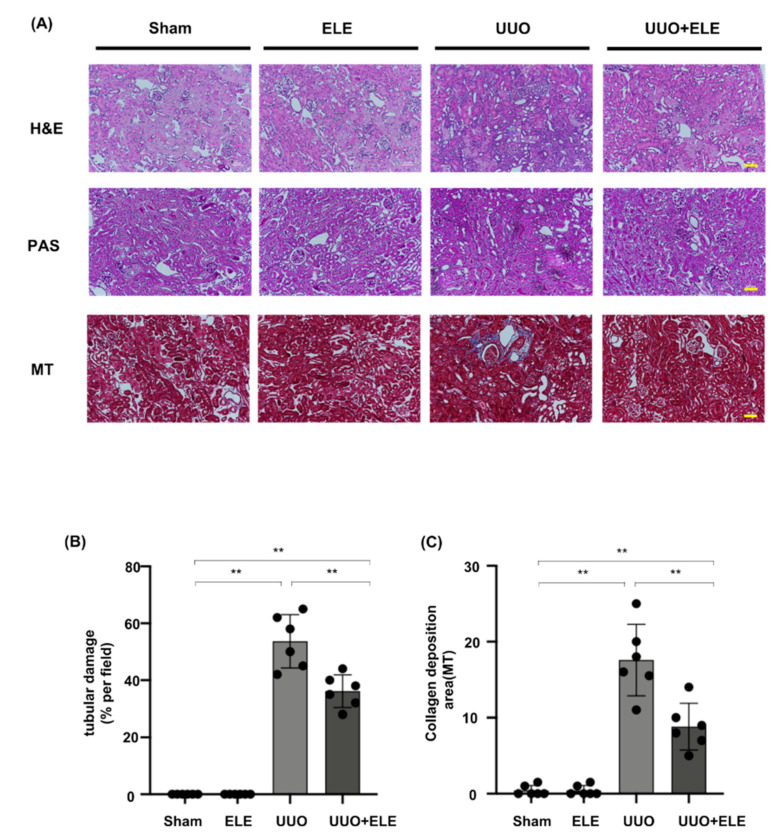
β-elemene ameliorated histopathological alterations in UUO mice. (**A**) Histological changes were assessed by H&E and PAS staining with typical interstitial tubular positive changes. Fibrosis was assessed by MT with typical interstitial fibers. Original magnification= ×20. Bar = 50 μm. (**B**) Kruskal-Wallis test for tubular damage, n = 6. ** *p* < 0.01, ns: no significance. (**C**) Statistical significance was presented as the mean ± SD, n = 6. ** *p* < 0.01, ns: no significance. Mice in the ELE and UUO+ELE groups were treated intraperitoneally with β-elemene at a dose of 40 mg/kg/d for 7 days, 24 h before and after surgery. All mice had their kidneys harvested on day 7. H&E, hematoxylin and eosin; PAS, Periodic Acid-Schiff stain; MT, Masson’s trichrome stain; ELE, β-elemene; UUO, unilateral ureteral obstruction.

We investigated the histological effects of β-elemene on renal interstitial fibrosis in UUO mice (Figure 1). H&E and PAS staining showed normal renal cortex in the Sham and ELE group. On the other hand, UUO mice showed partial tubular atrophy and interstitial expansion, interstitial fibrosis, and inflammatory cell infiltration in the obstructed kidney. PAS staining showed tubular brush border fracture and narrowed or even obstructed tubular lumen. However, these changes were significantly attenuated in the treated group of mice with β-elemene. MT staining showed significant ECM deposition in the UUO group, which was counteracted by treatment with β-elemene, reflecting a limited ECM extent and a lower degree of fibrotic deposition.

### 2.2. β-Elemene Attenuated the Expression of Fibrosis-Related Markers in UUO Mice

**Figure 2:**
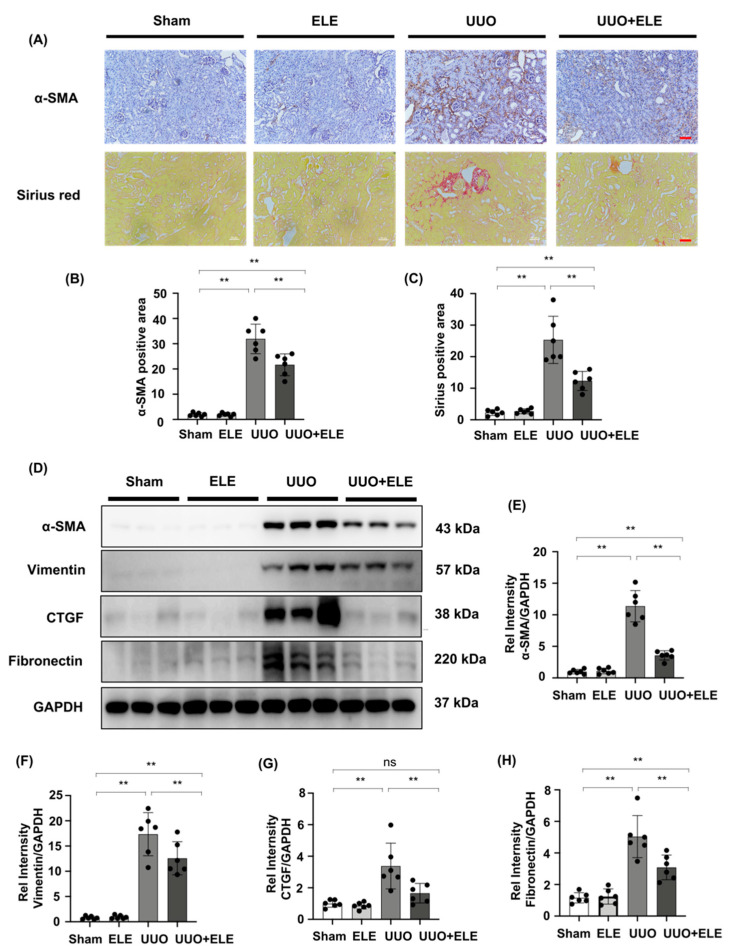
β-elemene attenuated the expression of fibrosis-related markers in UUO mice. (**A**) Expression of α-SMA in kidneys was detected by immunohistochemistry with α-SMA positive areas; Collagen I and III in kidney tissue sections were detected by Sirius red staining with Collagen positive areas. (**B**,**C**) Original magnification = ×20. Bar = 50 μm. Statistical significance was presented as the mean ± SD, n = 6. ** *p* < 0.01, ns: no significance. (**D**) Expression of α-SMA, vimentin, CTGF, and fibronectin in kidney tissues was detected by western blot and quantified by densitometry. (**E**–**H**) Statistical significance was presented as the mean ± SD, n = 6. ** *p* < 0.01, ns: no significance. Mice in the ELE and UUO+ELE groups were treated intraperitoneally with β-elemene at a dose of 40 mg/kg/d for 7 days, 24 h before and after surgery. All mice had their kidneys harvested on day 7. ELE, β-elemene; UUO, unilateral ureteral obstruction.

We analyzed α-SMA expression by immunohistochemistry and examined collagen I/collagen III deposition by Sirius staining (Figure 2A–C). Sham and ELE groups were largely devoid of deposition of these markers, and kidneys in the UUO group showed high expression of immunolabeling for α-SMA and collagen I/collagen III, which were attenuated in the UUO+ELE group. Further Western blot analysis confirmed that the fibrosis markers α-SMA, Vimentin, CTGF, and Fibronectin were largely absent in the sham and ELE group, expression of these marks was significantly elevated in the UUO group, and treatment with β-elemene significantly downregulated the expression of these markers (Figure 2D–H).

### 2.3. β-Elemene Inhibited TGF-β Stimulated Fibroblast Activation in NRK49F Cells

**Figure 3:**
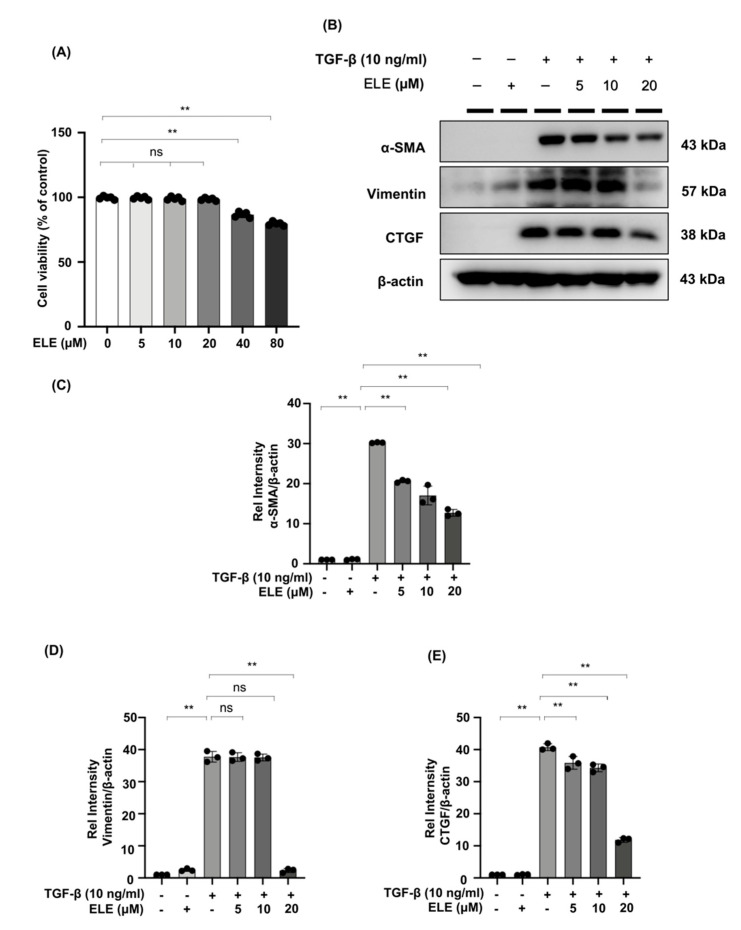
β-elemene inhibited TGF-β stimulated fibroblast activation in NRK49F cells. (**A**) Cells were treated with β-elemene for 24 h, and cytotoxicity assays were conducted. (**B**) Expression of α-SMA, CTGF, and vimentin in TGF-β stimulated NRK49F cells was detected by western blot and quantified by densitometry in a dose-dependent manner. Cells were pre-treated with β-elemene for 2 h after starvation with 0.5% FBS medium, treated with TGF-β (10 ng/mL) for 30 min, and incubated in 5% FBS medium for 24 h. (**C**–**E**) The Statistical significance was presented as the mean ± SD, n = 3. ** *p* < 0.01, ns: no significance. ELE, β-elemene.

We further investigated the effect of β-elemene on TGF-β stimulated NRK49F cells. We first examined the cytotoxic effect of β-elemene on NRK49F cells (Figure 3A). Our cell proliferation rate was almost 100% between 5–20 μM of β-elemene administration, and the normal growth state of the cells was not affected by β-elemene. Therefore, we chose 5–20 μM of β-elemene for the following experiments. It showed that α-SMA, vimentin, and CTGF protein expression in NRK49F cells were significantly increased in the TGF-β (10 ng/mL, 24 h) cultured group compared to the control group or the β-elemene treatment group alone. β-elemene pre-treatment has progressively decreased the expression of these markers in a dose-dependent manner (Figure 3B–E). These results suggest that β-elemene could inhibit TGF-β-induced activation of fibroblasts and expression of fiber markers in a dose-dependent manner in NRK49F cells.

### 2.4. β-Elemene Inhibited the Phosphorylation of JAK2/STAT3 and Smad3 and Suppressed the Expression of MyD88 in the UUO Model

**Figure 4:**
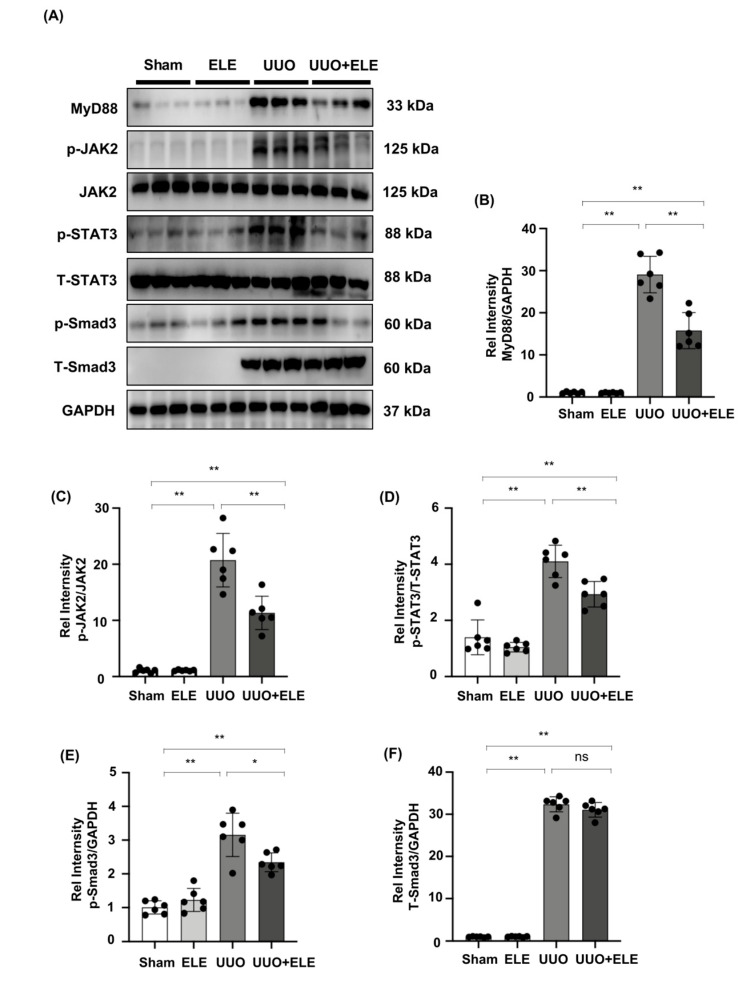
β-elemene inhibited the activation of JAK2/STAT3 signaling and reduced the expression of Smad3/MyD88 in the UUO model. (**A**) Expression of MyD88, p-JAK2, JAK2, p-STAT3, T-STAT3, p-Smad3, and T-Smad3 in the kidney model was detected by western blot and quantified by densitometry. (**B**–**F**) Statistical significance was presented as the mean ± SD, n = 6. * *p* < 0.05, ** *p* < 0.01, ns: no significance. Mice in the ELE and UUO + ELE group were treated intraperitoneally with β-elemene at a dose of 40 mg/kg/d for 7 days, 24 h before and after surgery. All mice had their kidneys harvested on day 7. ELE, β-elemene.

Increased phosphorylation of JAK2 and STAT3 was detected 7 days after UUO surgery, while the treatment with β-elemene reduced the activation of these signaling molecules. In addition, we found that Smad3 and MyD88 expression, as well as Smad3 phosphorylation, were enhanced in UUO kidneys compared to the sham and ELE group, and that these increases were diminished in the ELE+UUO group (Figure 4A–F).

### 2.5. β-Elemene Down-Regulated the Phosphorylation of JAK2/STAT3 and Smad3 and Inhibited the Expression of MyD88 in TGF-β-Stimulated NRK49F Cells

**Figure 5:**
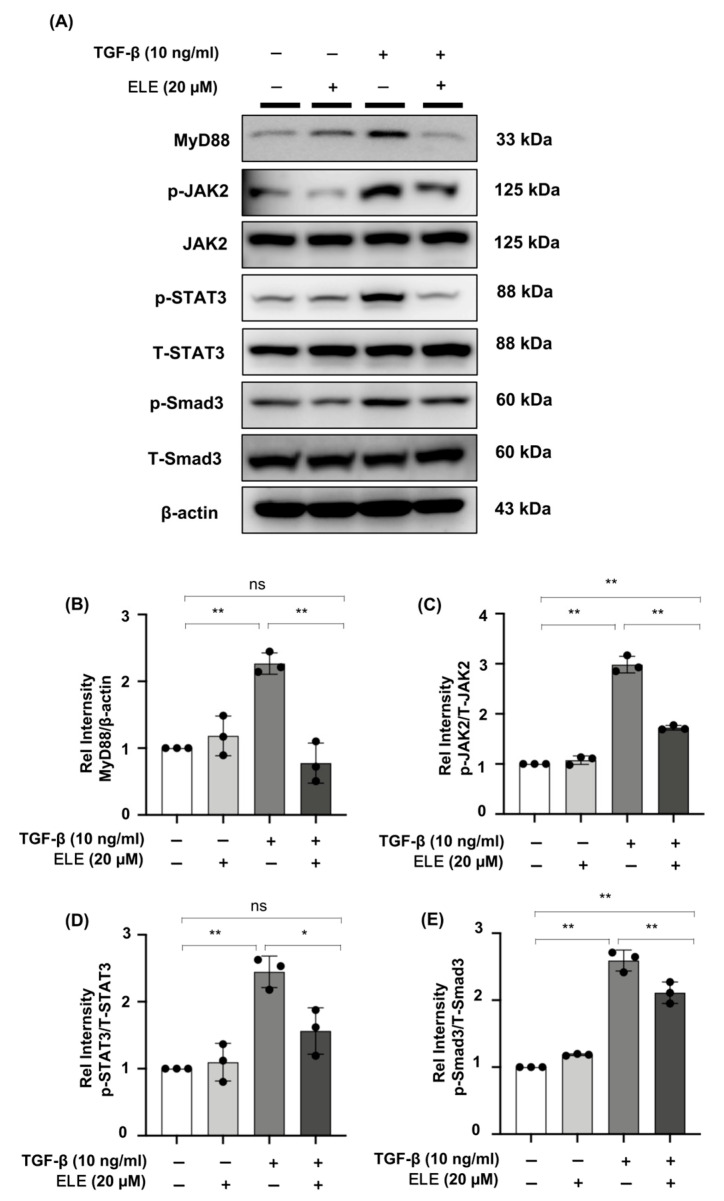
β-elemene down-regulated the phosphorylation of JAK2/STAT3 and Smad3 and inhibited the expression of MyD88 in TGF-β-stimulated NRK49F cells. (**A**) Expression of MyD88, p-JAK2, JAK2, p-STAT3, T-STAT3, p-Smad3, and T-Smad3 in TGF-β stimulated NRK49F cells was detected by western blot and quantified by densitometry. Cells were pre-treated with β-elemene for 2 h after starvation with 0.5% FBS medium and then treated with TGF-β (10 ng/mL) for 30 min. (**B**–**E**) Statistical significance was presented as the mean ± SD, n = 3. * *p* < 0.05, ** *p* < 0.01, ns: no significance. ELE, β-elemene.

Western blot experiments in NRK49F cells revealed an increase in MyD88 protein under TGF-β stimulation, and pre-treatment of β-elemene demonstrated a role by lowering its expression after 30 min. In TGF-β stimulated NRK49F cells, the expression of downstream proteins JAK2, T-STAT3, T-Smad3, and their phosphorylation products (p-JAK2, p-STAT3, p-Smad3) was examined further. After 30 min of TGF-β treatment, phosphorylated JAK2/STAT3 and Smad3 were increased in NRK49F cells, which was reduced by β-elemene co-treatment. The major changes in signaling were consistent with in-vivo experiments. These data imply that β-elemene may attenuate renal interstitial fibrosis by regulating the aforementioned JAK2/STAT3 signaling and Smad3/MyD88 activation (Figure 5A–E).

### 2.6. The STAT3 Inhibitor, NSC, and the Smad3 Inhibitor, SIS3 Reduced the Expression of Fibrotic Markers in TGF-β-Stimulated NRK49F Cells

**Figure 6:**
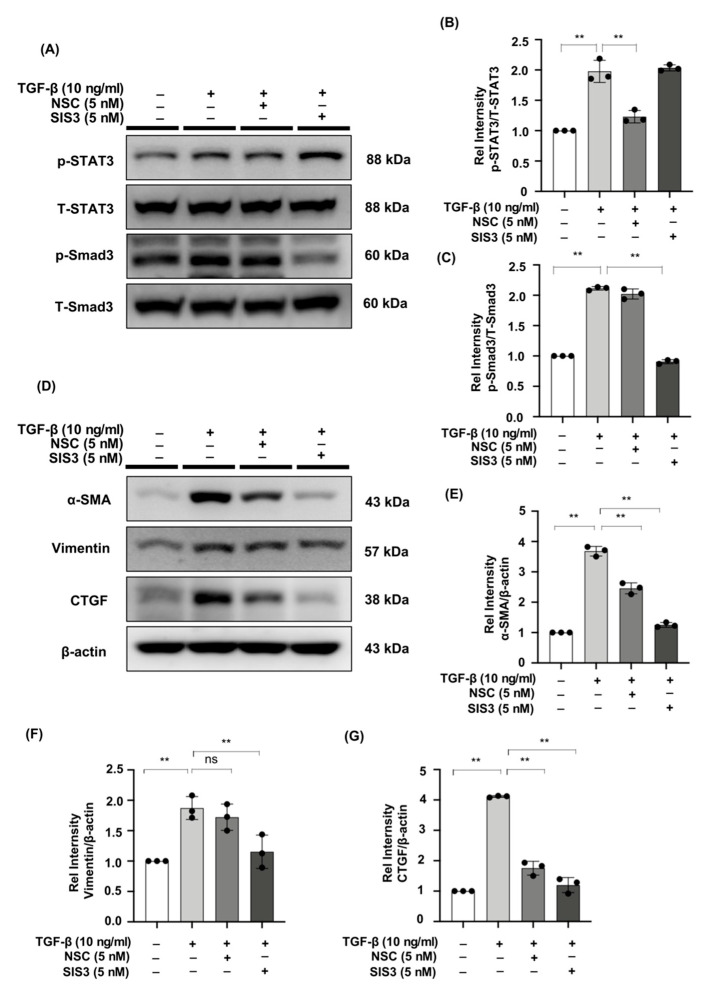
The STAT3 inhibitor, NSC, and the Smad3 inhibitor, SIS3, reduced the expression of fibrotic markers in TGF-β-stimulated NRK49F cells. (**A**) Expression of p-STAT3, T-STAT3, p-Smad3, and T-Smad3 in TGF-β-treated NRK49F cells with treatment of NSC and SIS3 was detected by western blot and quantified by densitometry. Cells were pre-treated with NSC and SIS3 for 2 h after starvation with 0.5% FBS medium and then treated with TGF-β (10 ng/mL) for 30 min. (**B**,**C**) Statistical significance was presented as the mean ± SD, n = 3. ** *p* < 0.01, ns: no significance. (**D**) Western blot demonstrated that NSC and SIS3 decreased the protein expression of α-SMA, vimentin, and CTGF in TGF-β stimulated NRK49F cells. NRK49F cells were treated with NSC or SIS3 for 2 h and then treated with TGF-β (10 ng/mL) for 30 min and incubated in a 5% FBS medium for 24 h. (**E**–**G**) Statistical significance was presented as the mean ± SD, n = 3. ** *p* < 0.01, ns: no significance.

Specific STAT3 phosphorylation inhibitor NSC and Smad3 phosphorylation inhibitor SIS3 were administered to NRK49F cells. TGF-β promoted STAT3 and Smad3 phosphorylation, while NSC and SIS3 specifically reversed p-STAT3 and p-Smad3 elevated expression (Figure 6A–C). Incubation with NSC and SIS3 inhibited the expression of α-SMA, vimentin, and CTGF (Figure 6D–G). The findings imply that TGF-β may activate the fibrotic process via STAT3 and Smad3 signaling.

### 2.7. MyD88 Silencing Suppresses STAT3 and Smad3 Phosphorylation in TGF-β Stimulated NRK49F Cells

**Figure 7:**
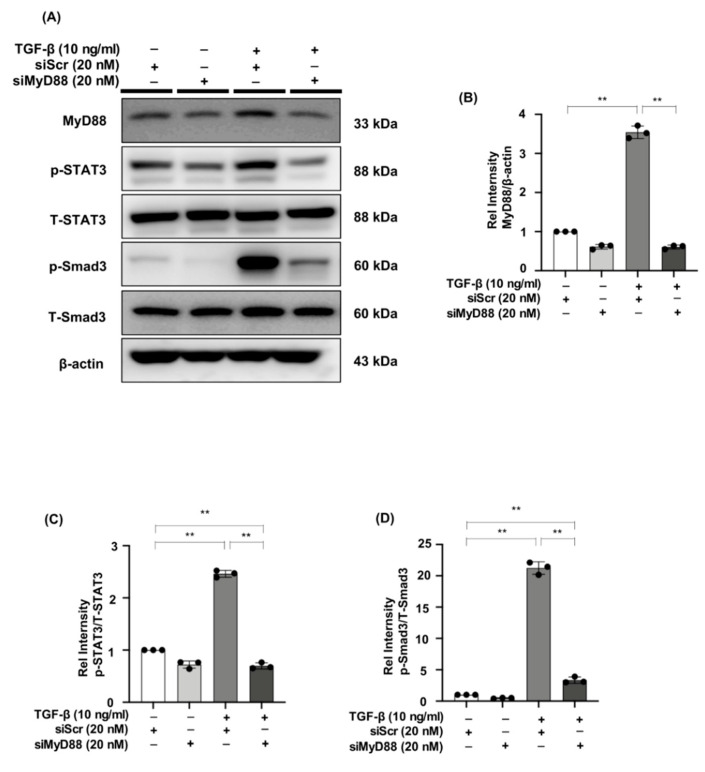
MyD88 silencing suppresses STAT3 and Smad3 phosphorylation in TGF-β stimulated NRK49F cells. (**A**) Western blotting revealed that p-STAT3 and p-Smad3 expression was blocked by siMyD88 transfection in TGF-β stimulated NRK49F cells. NRK49F cells were transfected with siMyD88 or siScr for 24 h in high-glucose DMEM without FBS, starved with 0.5% FBS medium, and then treated with TGF-β (10 ng/mL) for 30 min. Western blot demonstrated that MyD88 expression was significantly reduced by the specific siRNA. (**B**–**D**) Statistical significance was presented as the mean ± SD, n = 3. ** *p* < 0.01, ns: no significance.

We evaluated the effect of MyD88 knockdown on p-STAT3 and p-Smad3 expression in NRK49F cells. We observed decreased expression of MyD88 in TGF-β-induced NRK49F cells after successful transfection of siMyD88, p-STAT3 and p-Smad3 were elevated by TGF-β stimulation. Expression levels of the two markers were significantly down-regulated by siRNA of MyD88. However, the expression of T-STAT3 and T-Smad3 was not affected by siMyD88 (Figure 7A–D). These results suggested that the interaction of MyD88 and p-STAT3 or p-Smad3 may be another potential mechanism for β-elemene to reduce TGF-β-induced NRK49F cell fibrosis.

### 2.8. Co-Treatment of β-Elemene with Specific STAT3 Phosphorylation Inhibitor, Smad3 Phosphorylation Inhibitor, and siMyD88 Effectively Reduces Fibrosis in TGF-β Stimulated NRK49F Cells

**Figure 8:**
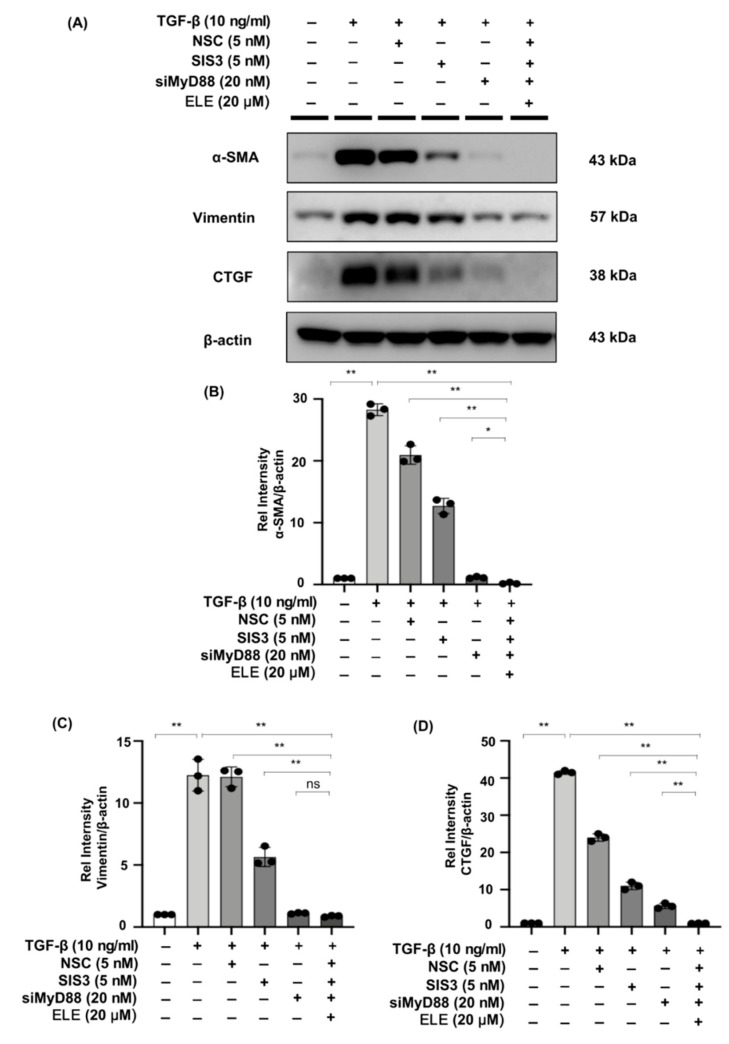
Co-treatment of β-elemene with specific STAT3 phosphorylation inhibitor, Smad3 phosphorylation inhibitor, and siMyD88 effectively reduces fibrosis in TGF-β stimulated NRK49F cells. (**A**) Western blotting revealed that α-SMA, vimentin, and CTGF expression was decreased by co-treatment of β-elemene with NSC, SIS3, and siMyD88 in TGF-β stimulated NRK49F cells. NRK49F cells were treated with NSC or SIS3 for 2 h and treated with TGF-β (10 ng/mL) for 30 min, and incubated in a 5% FBS medium for 24 h; NRK49F cells were transfected with siMyD88 for 24 h in high-glucose DMEM without FBS, starved with 0.5% FBS medium, then treated with TGF-β (10 ng/mL) for 30 min, and incubated in 5% FBS medium for 24 h; NRK49F cells were transfected with siMyD88 for 24 h in high-glucose DMEM without FBS, starved with 0.5% FBS medium, and then treated with NSC, SIS3 and β-elemene for 2 h before treated with TGF-β (10 ng/mL) for 30 min, and incubated in 5% FBS medium for 24 h. (**B**–**D**) Statistical significance was presented as the mean ± SD, n = 3. * *p* < 0.05, ** *p* < 0.01, ns: no significance.

We evaluated the effects of β-elemene with specific STAT3 phosphorylation inhibitor NSC, Smad3 phosphorylation inhibitor SIS3, and siMyD88 on TGF-β stimulated fibrosis-related markers in NRK49F cells. In the TGF-β stimulated cells treated with NSC, SIS3, siMyD88, and β-elemene together, the expression of α-SMA and CTGF was lower than that in each of the TGF-β-induced groups treated with NSC, SIS3, siMyD88 only, and the expression of vimentin was lower than in the TGF-β-induced groups treated with NSC or SIS3 only, while it was not significantly different from that in the TGF-β + siMyD88 group (Figure 8A–D).

**Figure 9:**
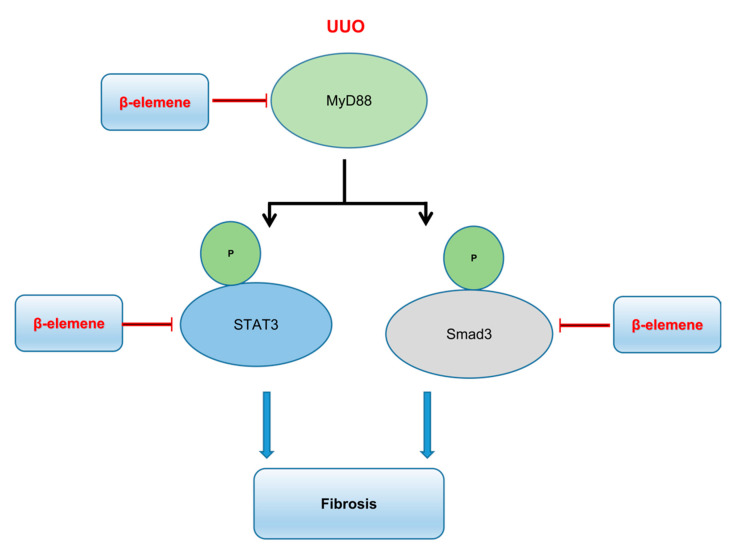
Schematic diagram of the mechanism of action of β-elemene on the therapeutic effect of fibrosis in UUO mice.

The above results show that β-elemene treatment attenuates kidney fibrosis markers through its influence on STAT3, Smad3, and MyD88-dependent fibrotic signaling. Figure 9 is a schematic diagram illustrating the results of the present study.

## 3. Discussion

Chronic kidney disease (CKD) is not only increasingly becoming a global epidemic posing huge social and economic problems, it is also an independent risk factor for high mortality from chronic diseases such as diabetes, hypertension, and cardiovascular disease [34,35]. Almost all acute and chronic kidney injuries will progress to CKD and eventually to ESRD [36]. The underlying pathology of both CKD and ESRD is renal fibrosis [36]. Although many potential therapeutic targets have been identified, there are no specific treatment options effective in improving renal fibrosis [37,38]. In this study, we found that β-elemene attenuated renal interstitial fibrosis and down-regulated ECM protein expression in UUO mice by inhibiting STAT3 and Smad3 signaling via suppressing MyD88 expression, suggesting that β-elemene is effective in the treatment of renal fibrosis.

Hong et al. demonstrated that β-elemene ameliorates esophageal fibrosis by reducing the expression of α-SMA, collagen I, and fibronectin [39]. We demonstrated that β-elemene similarly ameliorated renal tubular injury and fibrosis in the UUO model. β-elemene effectively inhibited the high expression of molecules that play a major role in renal tubular interstitial fibrosis, including α-SMA, CTGF, vimentin, and fibronectin, thereby reducing the accumulation of ECM in renal tissue. In vitro experiments, TGF-β induced cellular fibrosis in NRK49 cells, leading to an increase in fibroblast markers, such as α-SMA, vimentin, and CTGF. Pre-treatment of cells with β-elemene resulted in a significant downregulation of these fibrosis-related proteins in a dose-dependent trend, which together with in vivo observations, suggests the therapeutic potential of β-elemene for renal fibrosis.

Smad3 is pathogenic in the context of TGF-β/Smad signaling, and it has been proven that Smad3 overexpression and phosphorylation not only increase high protein levels in kidney fibrosis caused by diverse etiologies but also block TGF-β induced ECM reduction [40,41,42]. Zhang et al. showed that β-elemene blocked the epithelial-mesenchymal transition of human breast cancer cell line MCF-7 by inhibiting the expression of Smad3 [43]. In the present experiments, β-elemene significantly reduced the phosphorylation level of Smad3 in UUO and TGF-β stimulated NRK49F cells. Previous studies have shown that mice lacking Smad3 have reduced EMT and collagen accumulation and reduced renal tubular interstitial fibrosis after unilateral ureteral obstruction [44,45]. Another study showed that interventions targeting intracellular phosphorylation of Smad2/3 proteins also reduced TGF-β-induced fibrosis [46]. Using SIS3, an inhibitor of selective Smad3, we demonstrated that inhibition of p-Smad3 production in NRK49F cells significantly reduced ECM protein expression. It can be inferred that β-elemene might serve as another Smad3 inhibitor to improve renal interstitial fibrosis by targeting this signaling factor.

In vitro studies by Chakraborty et al. showed that the phosphorylation level of STAT3 in TGF-β stimulated fibroblasts could be reduced using JAK2 inhibitors [26]. Meanwhile, several studies have shown that increased STAT3 activation is involved in UUO-induced renal fibrosis [47,48]. In our study, β-elemene treatment inhibited the phosphorylation of JAK2/STAT3 signaling in the kidney in UUO mice, and TGF-β stimulated NRK49F cells. Notably, the expression levels of α-SMA, vimentin, and CTGF in NRK49F cells were significantly reduced after inhibiting STAT3 phosphorylation signaling using NSC, indirectly indicating the inhibitory effect of β-elemene on STAT3 ultimately affects the ECM process.

The association of the MyD88 signaling factor with fibrosis has been reported [49,50]. In our previous report, it was demonstrated that silencing of MyD88 inhibits TGF-β stimulated fibrosis in NRK49F cells and reduces the protein expression levels of α-SMA, CTGF, and vimentin [51]. Notably, Xiu Qiong Fu et al. showed that TLR4 ligands activate STAT3 through MyD88 in melanoma cells and that knockdown of MyD88 reduced the level of STAT3 phosphorylation without affecting total STAT3 protein levels [52]. Signal crosstalk between MyD88 and the Smad family has been reported involving Smad4 and Smad6, etc. [53,54], but there are few studies on whether there is an association between MyD88 and Smad3 [55]. In the present study, β-elemene significantly inhibited MyD88 expression in UUO mice, and TGF-β stimulated NRK49F cells. In subsequent experiments, siRNA targeting MyD88 inhibited the phosphorylation levels of STAT3 and Smad3. Furthermore, we observed that co-treatment of β-elemene with NSC, SIS3, and siMyD88 resulted in a more significant decrease in the fibrotic markers. Taken together, β-elemene inhibited renal interstitial fibrosis, perhaps by targeting STAT3-Smad3-MyD88 crosstalk and signaling.

In conclusion, this experimental study shows that β-elemene reduces renal fibrosis by targeting JAK2/STAT3, Smad3, and Myd88 signaling factors and may serve as a potent inhibitor of STAT3 and Smad3 in the future. Meanwhile, we elucidated a novel cross-regulation between MyD88 and STAT3 or Smad3, thus reaffirming the plausibility of MyD88 as a potential therapeutic target for renal fibrosis. These findings may help identify new signaling pathways associated with renal fibrosis and help unravel the pathogenesis of CKD in the near future.

## 4. Materials and Methods

### 4.1. Reagents and Antibodies

β-elemene (63965) was purchased from Sigma-Aldrich (St. Louis, MO, USA). The primary antibodies used for immunoblotting were as follows: anti-α-SMA (A2547; Sigma); anti-fibronectin (SC-71114), anti-β-actin (SC-47778), anti-CTGF (SC-14939) came from Santa Cruz Biotechnology (Dallas, TX, USA); anti-GAPDH (AM4300; Ambion, Austin, TX, USA); anti-T-Smad3 (#9513S), anti-p-Smad3 (#9520S), anti-T-STAT3 (#12643), anti-p-STAT3 (#9131S), anti-T-JAK2 (#3230S), anti-p-JAK2 (#3776S) anti-MyD88 (#4283S) and anti-vimentin (#5741S) were purchased from Cell Signaling Technology (Danvers, MA, USA).

### 4.2. Animal Experiments

Eight-week-old male C57BL/6 mice (20–25 g) were purchased by Samtako (Osan, South Korea). We randomized the mice into four groups: sham-operated group (n = 6), β-elemene-only group (following called ELE group) (n = 6), UUO group (n = 6), β-elemene treated UUO group (following called UUO + ELE group) (n = 6). The peritoneal cavity was fully exposed by a median abdominal incision, anesthetized with ketamine (50 mg/kg; Yuhan, Seoul, Korea). A 4-0 silk ligature was subsequently placed in the proximal left ureter, following our previous report [56]. The sham group received the same surgical procedure without ligation of the ureter. Mice in the ELE and UUO + ELE groups were treated intraperitoneally with β-elemene at a dose of 40 mg/kg/d for 7 days, 24 h before and after surgery [57]. All mice had their kidneys harvested on day 7. All animal experiments were approved by the Animal Care Regulations (ACR) Committee of the All South University School of Medicine (CNUH IACUC-22006, 19 April 2019). Our protocols conformed to the institutional guidelines for the care and use of laboratory animals.

### 4.3. Cell Culture and Treatment

NRK49F cells (American Type Culture Collection, Manassas, VA, USA) were cultured in DMEM high glycemic medium (Welgene, Daegu, Korea) containing 5% fetal bovine serum (FBS) and 1% streptomycin/penicillin. The incubator was adjusted to 37 °C and 5% CO_2_ atmosphere. After 70–80% confluence, cells were starved with 0.5% FBS medium for 24 h, pre-treated with β-elemene for 2 h, and then incubated with TGF-β (10 ng/mL) (Pepro Tech, Rocky Hill, NJ, USA) for 30 min or 24 h. β-elemene was dissolved in dimethyl sulfoxide (DMSO) as a 40 mM stock solution and stored at −20 °C.

### 4.4. Histology

One-third of the kidney tissue sections were separated, fixed, embedded in paraffin, and sectioned. Sections were stained with hematoxylin and eosin (H&E) after dewaxing with gradient ethanol solution. After 5 min of staining with Gill’s hematoxylin, kidney slices were fractionated with 0.3% acidic alcohol and incubated in eosin and filosin for 2 min. Finally, the slides were dehydrated and mounted.

Periodic acid-Schiff (PAS) staining was done following the manufacturer’s instructions. Deparaffinize and hydrate the sections; submerge the slide in PAS solution for 5−10 min; wash 4 times with water; immerse the slide in Schiff’s solution for 15−30 min and rinse; and finally stain the slide with Hematoxylin for 2–3 min and rinse. Incubate for 30 s in Bluing Reagent, then rinse and incubate for 2 min in Light Green Solution. Injury of the renal tubules was defined according to our previous study. Reversible injury of tubules showed as the absence of brush border. Irreversible injury included tubular dilatation, tubular cell necrosis and apoptosis, and basement membrane exfoliation, etc.

For Masson’s trichrome (MT) staining, sections were degreased sequentially in 100%, 95%, and 70% alcohol, refixed in Bouin’s solution at 56 °C for 1 h to improve staining quality, Weigert’s iron hematoxylin solution was added, sections were incubated in Biebrich scarlet/acidic violet-red solution for 10−15 min, fractionated in the phosphotungstic acid-phosphomolybdic acid solution for 10 min and transferred directly to blue aniline solution for 10 min. The slices were then treated in acetic acid for 2 min before being immediately dehydrated with ethanol and xylene. Blue, black, and red dyes were used to stain collagen deposits, nuclei, and muscle fibers, respectively.

### 4.5. Immunohistochemistry and Immunofluorescence Staining

Primary α-SMA antibody (A2547; Sigma) and HRP anti-mouse IgG secondary antibody were used for immunohistochemical staining (Dako, Glostrup, Denmark). The protocol for Sirius red staining utilized Picro Sirius Red Stain Kit (Connective Tissue Stain) (ab150681).

Visual fields were chosen randomly from digital images for each section at a magnification of 20×. A Nikon microscope was used to capture the stained sections (Tokyo, Japan). ImageJ software was used to perform quantitative analysis on the stained area.

### 4.6. Cytotoxicity Assay

NRK49F cells were cultivated at a density of 1 × 10^4^ cells/mL in a volume of 100 μL/well in 96-well plates and allowed to attach for 24 h. The cells were starved for 24 h in a 0.5% FBS medium before being placed into the correct dosage of β-elemene 5, 10, 20, 40, and 80 μM for another 24 h. Cell viability was evaluated after incubating the cells for 0.5–2 h at 37 °C with 10 uL of WST solution EZ-Cytox-1000 (Dogen). A microplate reader was used to measure the absorbance at 450 nm (Bio-Tek Instruments, Winooski, VT, USA).

### 4.7. siRNA Knockdown

MyD88 rat siRNA (Santa Cruz Biotechnology) was used to interfere with MyD88 (Rockville, MD, USA. Cat. SC-106986). Cells were transfected with the required concentration of MyD88 siRNA (20 nM) (following siMyD88) using the DhamaFECT 1 transfection reagent according to the manufacturer’s instructions. For comparison, cells transfected with scrambled siRNA (Santa Cruz Biotechnology, Cat. SC-37007; Dallas, TX, USA) (following siScr) were performed as controls.

### 4.8. Western Blotting

RIPA buffer was used to lyse total protein from kidneys or cells frozen in liquid nitrogen (Thermo Scientific, Waltham, MA, USA). Tissue/cell debris was discarded and the supernatant was collected after short centrifugation (13,000× *g*). The BCA Protein Assay kit (Thermo Scientific) was used to assess protein concentration according to the manufacturer’s instructions. SDS-PAGE gels were used to electrophorese equal volumes of lysates (Invitrogen). Proteins were separated, transferred to PVDF membranes, and blotted with particular antibodies. ImageJ was used to measure the densitometry values.

### 4.9. Statistical Analysis

Parametric variables were presented as the mean ± SD. Multiple group comparisons were performed using one-way ANOVA and Tukey’s honestly significant difference (HSD) post hoc test. Nonparametric variables were presented as medians with interquartile (25th and 75th percentiles) ranges for continuous variables. Kruskal-Wallis test with Dunn’s multiple comparison post-hoc testing for tubular damage nonparametric data. Results with *p* < 0.05 were considered statistically significant.

## 5. Conclusions

In conclusion, we demonstrated that β-elemene attenuated renal interstitial fibrosis in unilateral ureteral obstruction (UUO) mice and in TGF-β-stimulated rat renal interstitial fibroblasts (NRK49F cells). Notably, β-elemene ameliorated the renal fibrotic process by specifically interfering with MyD88, thereby attenuating the phosphorylation levels of STAT3 and Smad3. This study confirmed that β-elemene is a promising candidate for the treatment of renal interstitial fibrosis. It can be predicted that β-elemene may become a targeted drug for the treatment of CKD in the future.

## Data Availability

The data is available on request from the corresponding author.
